# Specific In Vivo Labeling of Tyrosinated α-Tubulin and Measurement of Microtubule Dynamics Using a GFP Tagged, Cytoplasmically Expressed Recombinant Antibody

**DOI:** 10.1371/journal.pone.0059812

**Published:** 2013-03-28

**Authors:** Lynne Cassimeris, Laurence Guglielmi, Vincent Denis, Christian Larroque, Pierre Martineau

**Affiliations:** 1 Department of Biological Sciences, Lehigh University, Bethlehem, Pennsylvania, United States of America; 2 IRCM, Institut de Recherche en Cancérologie de Montpellier, Montpellier France; 3 INSERM, U896, Montpellier, France; 4 Université Montpellier1, Montpellier, France; 5 CRLC Val d’Aurelle Paul Lamarque, Montpellier, France; The Beatson Institute for Cancer Research, United Kingdom

## Abstract

GFP-tagged proteins are used extensively as biosensors for protein localization and function, but the GFP moiety can interfere with protein properties. An alternative is to indirectly label proteins using intracellular recombinant antibodies (scFvs), but most antibody fragments are insoluble in the reducing environment of the cytosol. From a synthetic hyperstable human scFv library we isolated an anti-tubulin scFv, 2G4, which is soluble in mammalian cells when expressed as a GFP-fusion protein. Here we report the use of this GFP-tagged scFv to label microtubules in fixed and living cells. We found that 2G4-GFP localized uniformly along microtubules and did not disrupt binding of EB1, a protein that binds microtubule ends and serves as a platform for binding by a complex of proteins regulating MT polymerization. TOGp and CLIP-170 also bound microtubule ends in cells expressing 2G4-GFP. Microtubule dynamic instability, measured by tracking 2G4-GFP labeled microtubules, was nearly identical to that measured in cells expressing GFP-α-tubulin. Fluorescence recovery after photobleaching demonstrated that 2G4-GFP turns over rapidly on microtubules, similar to the turnover rates of fluorescently tagged microtubule-associated proteins. These data indicate that 2G4-GFP binds relatively weakly to microtubules, and this conclusion was confirmed in vitro. Purified 2G4 partially co-pelleted with microtubules, but a significant fraction remained in the soluble fraction, while a second anti-tubulin scFv, 2F12, was almost completely co-pelleted with microtubules. In cells, 2G4-GFP localized to most microtubules, but did not co-localize with those composed of detyrosinated α-tubulin, a post-translational modification associated with non-dynamic, more stable microtubules. Immunoblots probing bacterially expressed tubulins confirmed that 2G4 recognized α-tubulin and required tubulin’s C-terminal tyrosine residue for binding. Thus, a recombinant antibody with weak affinity for its substrate can be used as a specific intracellular biosensor that can differentiate between unmodified and post-translationally modified forms of a protein.

## Introduction

Localization of proteins within cells typically relies on either expression of fluorescently tagged proteins or by labeling proteins with specific antibodies. While each method is powerful, each has limitations. Expression of fluorescently tagged proteins allows dynamic processes to be followed in living cells, but often represents over-expression of the protein of interest and the large size of the fluorescent protein can interfere with the function. For example, gap junctions assembled from connexin-43 tagged at its C-terminus are much larger than gap junction plaques assembled from untagged connexin-43; the larger plaque size is due to the loss of binding between GFP-tagged connexin-43 and zonula occludens-1 [1]. Expression of fluorescently tagged proteins also does not allow discrimination between different post-translational modifications to that protein. On the other hand, antibodies recognizing specific post-translational modifications are available, but are typically only used after cell fixation, requiring information from cell populations to extrapolate the steps in a dynamic process. Combining the specificity of antibodies with expression of fluorescently tagged proteins in the mammalian cell cytoplasm has become possible through the use of hyperstable recombinant antibodies optimized for expression under reducing conditions [2], [3].

The most widely used recombinant antibody format is expression of a single chain polypeptide encompassing the heavy and light chain variable regions from immunoglobin (termed single chain variable region, scFv). Phage display is used to screen these libraries and has allowed selection of scFv specific for protein conformation (e.g., tubulin bound to GTP [4]). Typically scFv show poor solubility in the reducing environment of the cytosol and are non-functional under these conditions, limiting their use to post-fixation protein localizations, similar to conventional antibodies generated in animals [5], [6]. To extend application of scFv for intracellular expression, we constructed a synthetic library of scFv’s that are expressed in the cytoplasm of bacteria and mammalian cells [3]. These scFv are often termed intrabodies because of their intracellular expression. Screening the library identified several scFv’s that recognize tubulin [3], [7], including one that is soluble in both E. coli and mammalian cells (2G4) and a second (2F12) that is soluble in E. coli but present as aggregates when expressed in mammalian cells [7].

Heterodimers of α and ß tubulin polymerize to form microtubules, polymers responsible for a number of cellular functions including chromosome movement in mitosis, vesicle transport, intracellular organization and as scaffolds for components of signal transduction pathways [8], [9], [10]. Microtubules are dynamic polymers and typically undergo dynamic instability, continually switching between states of subunit addition and loss from the plus end of the microtubule, with abrupt transitions between states [11], [12]. This dynamic turnover allows the microtubule cytoskeleton to reorganize rapidly in response to cues, including entry into mitosis. Under certain conditions, some microtubules become stabilized (become non-dynamic) and are targets for post-translational modifications to the tubulin subunits within these non-dynamic microtubules [9]. Thus, tubulin post-translational modifications, particularly α-tubulin acetylation or detyrosination, serve as markers to differentiate between dynamic and non-dynamic microtubule subpopulations, although the modifications themselves do not confer stability [9], [13]. Detyrosination and acetylation do not necessarily mark the same microtubule subsets within cells, and the type of modification present can also change over time [14], highlighting the dynamic nature of tubulin posttranslational modifications. Antibodies specific to acetylated or detyrosinated tubulins have been used to localize non-dynamic microtubules (e.g. [15], [16]), however, their use has been restricted to fixed cells.

Here we report the intracellular expression of a GFP tagged recombinant scFv, 2G4-GFP, that is soluble in the cytoplasm of cells and co-localizes with microtubules without disrupting microtubule polymerization dynamics. Fluorescence recovery after photobleaching and in vitro microtubule co-pelleting experiments indicate that 2G4-GFP binds microtubules relatively weakly, with an affinity for microtubules similar to several microtubule-associated proteins (MAPs). This scFv specifically recognizes tyrosinated, but not detyrosinated, α-tubulin. We suggest that visual screens of GFP-tagged scFvs could be used to isolate scFv recognizing specific post-translationally modified states of a protein of interest, and that scFvs showing a localization pattern in living cells similar to antibody localization in fixed cells are likely to bind with sufficiently weak affinity to mark a protein without compromising its function. Such scFv could be combined with protein conformation-sensitive scFv [2] to define simultaneously changes in protein localization, modification and conformation in living cells.

## Methods

### Cell Lines and Transfections

Hela, LLCPK and MEF cells, obtained from ATCC, were maintained as described previously [17], [18]. Cells were transfected with a plasmids (1 µg per 35 mm dish) for expression of GFP-tau (3 microtubule binding repeats, tau accession number BC000558 in pcDNA 3.1; [19], 2G4-GFP or 2F12-GFP [7], or GFP alone (accession number U55762), using X-tremeGENE HP DNA Transfection Reagent (version 1.0; Roche Diagnostics, Indianapolis, IN) according to the manufacturer’s protocol. Cells were examined 20 - 24 h after transfection. For comparison to 2G4-GFP expressing cells, an LLCPK cell line stably expressing GFP-α-tubulin was also used to measure microtubule dynamics [20].

### Immunofluorescence

Cells were fixed in −20°C methanol/EDTA and stained as described previously [16]. Antibodies used included mouse anti-alpha tubulin (1∶1000 dilution, clone B-5-1-2; Sigma), mouse anti-acetylated α-tubulin (1∶1000 dilution, clone 6-11B-1; Sigma), rabbit anti-detyrosinated α-tubulin (1∶50 dilution, Millipore Inc.), mouse anti-EB1 (1∶50 dilution, BD Biosciences), rabbit anti-TOGp (1∶1000; [21], and mouse anti-CLIP-170 (mixture of 4D3/2D6 diluted 1∶100; [22]). In some experiments to localize CLIP-170, a two step fixation procedure of 20 min incubations in −20°C methanol/EDTA followed by 4% paraformaldehyde in 4.2% sucrose/PBS [23] was used. Goat anti-mouse or anti-rabbit Alexa 568 antibodies (1∶50 - 1∶200 dilutions; Invitrogen) were used to detect primary antibodies. These goat secondary antibodies did not recognize the expressed scFv. Coverslips were mounted in Vectashield (Vector Laboratories, Inc.) and examined by confocal microscopy as described previously [24].

### ScFv Purification

For experiments exploring scFv binding to purified tubulin, scFv’s tagged with 6x His were expressed in E. coli and purified as described previously [3].

### GST-Tubulin Fusion Protein Expression in E. coli

Genes encoding human TUBA1A and TUBB were inserted in frame at the 3′ extremity of the GST gene in the pGEX-4T1 vector. Deletion of the C-terminal tyrosine of the TUB1A1 gene was performed by PCR. All inserts were fully sequenced. TG1 cells containing plasmids were grown in LB/ampicillin medium to an OD 600nm of 0.4, then 1 mM of IPTG was added and the culture continued for 3 h at 37°C with shaking. Cells were pelleted and resuspended in the same volume of Laemmli buffer (0.01% bromophenol blue, 5% β-mercaptoethanol, 2% SDS, 10% glycerol, 62.5 mM Tris-HCl pH 6.8), and boiled. Proteins were separated on a 10% SDS-PAGE gel and transferred on a nitrocellulose membrane. The membrane was saturated in PBSTM (PBS, Tween 0.1%, Non-fat milk 2%) for 1 h, incubated with purified 2G4 (10 µg/ml) for 2 h in the same buffer. Bound scFv was revealed using an HRP-coupled anti-His-tag antibody (HIS-1, monoclonal; Sigma H1029), which recognizes the His tag on bacterially expressed scFv. A rabbit anti-GST polyclonal antibody was used to confirm equal loading of GST-tubulin fusion proteins. Expression of the detyrosinated mutant was confirmed using a rabbit antibody specific for detyrosinated a-tubulin [25]. The signal was revealed using enhanced chemiluminescence and detected with a camera (G:BOX Chemi, Syngene, Cambridge, UK).

### Immunoblotting of Tissue Extracts

Soluble porcine brain extracts were separated on a 10% SDS-PAGE gel (1 µg/lane) and then transferred to a nitrocellulose membrane. Membranes were saturated in PBSTM for 1 h and incubated with 13R4 (10 µg/ml), 2G4 (10 µg/ml), 2F12 (2 µg/ml), a rabbit polyclonal serum raised against purified tubulin (a kind gift of Ned Lamb), or a monoclonal anti-tubulin (clone Tub2.1; Sigma T4026). Bound antibodies were revealed with a suitable HRP-coupled secondary antibody (anti-His-tag Sigma H1029 for the 3 scFv’s) and enhanced chemiluminescence detected with a Hyperfilm.

### Tubulin Purification and Assembly

Pig brains were obtained from the Abattoir of Aves (France) under supervision of local employees and the staff veterinarian. Tubulin was isolated from porcine brains by a combination of two cycles of microtubule assembly/disassembly, followed by phosphocellulose chromatography [26]. Tubulin polymerization was monitored as previously described [27]. Briefly, 12 µM purified tubulin was polymerized in the presence or absence of GTP and 0.67 µM of each purified scFv in a total volume of 200 µl. Under the conditions used, about 50% of the tubulin was polymerized into microtubules and no effect of the scFv on tubulin polymerization was detected. To obtain different ratios of tubulin/microtubules, 20 µM Taxol was included in some reaction mixtures and microtubules polymerized at 25°C and 37°C. The preparations were applied onto a 40% sucrose cushion centrifuged at 400,000 × g for 1 h at 25°C in a SW60 rotor. The pellet was resuspended in a volume equal to the supernatant and proteins resolved on an 11% SDS-PAGE gel. Proteins were detected by Coomassie blue staining.

### Live Cell Imaging and Photobleaching

For observations of living cells expressing fluorescently tagged proteins, cells were plated onto either glass bottom dishes (MatTek Corp., Ashland, MA) or 30 mm round coverslips, and transferred to high glucose DMEM lacking phenol red and supplemented with 10 mM HEPES pH 7.3, 10% fetal bovine serum, 1 mM sodium pyruvate just prior to imaging. For photobleaching experiments, 30 mm round coverslips were mounted into a POCmini chamber (PeCon GmbH, Erbach, Germany). The POCmini chamber was maintained at 37°C on a Zeiss LSM confocal microscope stage through use of stage and objective heaters (PeCon GmbH, Erbach, Germany). Cells on glass bottom dishes were imaged on a swept field confocal system (described below); temperature was maintained at 37°C by an environmental chamber (InVivo Scientific, St. Louis, MO).

Photobleaching was performed using a Zeiss LSM510META confocal microscope as described previously [24]. Briefly, cells were imaged using a 63X/1.4 NA planapo objective on a Zeiss Axiovert 200M. The LSM software was used to control laser illumination and image acquisition. GFP was imaged using a 488 nm argon ion laser; the 30 mW laser was run at approximately 50% current, and transmission reduced to 1% for image acquisition. Images (1.1 µm optical section, 8 bit, either 1024 x 1024 or 512 x 512 pixels) were acquired at 1 - 4 s intervals for approximately 50 s total time. Two images were collected prior to photobleaching and then a rectangular region of approximately 2 µm x 5 - 10 µm was photobleached by illuminating this region with 4 scans from the 488 nm laser line at 100% transmission. Increasing the number of photobleaching scans to 20 or 60 iterations did not affect the half time of recovery.

Microtubule dynamics, as marked by 2G4-GFP or GFP-α-tubulin, were imaged using a swept field confocal system (Prairie Technologies, Middleton, WI) mounted to a Nikon Eclipse Ti inverted microscope equipped with a 100X/1.4 NA planapo objective. A 488 nm solid-state sapphire laser (30 mW, used at 5 - 10% transmission) was used to excite GFP. Images were collected using a 35 mm slit aperture and an exposure time of 900 ms. Images were projected through a 1.5X optivar to a Photometrics QuantEM 512SC camera. Image acquisition was controlled by NIS Elements AR software version 3.0 (Nikon Imaging, Melville NY). Images were exported as TIFF files and imported into MetaView imaging software (Molecular Devices, Downingtown, PA) for analysis of microtubule length changes over time [17], [28]. Time-lapse sequences were advanced frame by frame and the position of a microtubule end, as marked by 2G4-GFP or GFP- α-tubulin, was tracked over time. The lengths of microtubules at time points were exported to Microsoft Excel and used to calculate the parameters of dynamic instability as described previously [28]. Transition frequencies were calculated as the number of catastrophes or rescues per unit time (s^−1^) [28]. Dynamicity was calculated for each microtubule by adding the total growth length and the total shortening length and dividing by the total imaging time [28].

### Image Analysis

The rate of fluorescence recovery after photobleaching was calculated from integrated fluorescence intensity measurements within either the bleached region or along a line on single microtubules. Zeiss LSM software was used to measure the integrated fluorescence intensity. Data were exported to an Excel spreadsheet for further calculations. Fluorescence intensities were normalized by setting the average pre-bleach fluorescence to 100. Fluorescence intensity within an unbleached area (or from a line along an unbleached microtubule) was used to correct for photobleaching caused by image acquisition. Plots are shown as normalized, corrected fluorescence over time. Fluorescence recovery rate was calculated from an exponential function, as described previously [19]: F_inf_ – F(t) = [F_inf_ – F(0)]e^−kt^, where F_inf_ is the fluorescence intensity after maximum recovery, F(t) is the fluorescence intensity at each time point and F(0) is the pre-bleach fluorescence intensity. The first order rate constant, k, was calculated from plots of ln[F_inf_ - F(t)] versus time and used to calculate the half time of recovery (t_1/2_ =  ln2/k).

### Statistics

All quantitative data are presented as mean ± SD. Statistical significance was determined using unpaired t-tests.

## Results

### 2G4-GFP Colocalizes with Microtubules in Mammalian Cells

**Figure 1:**
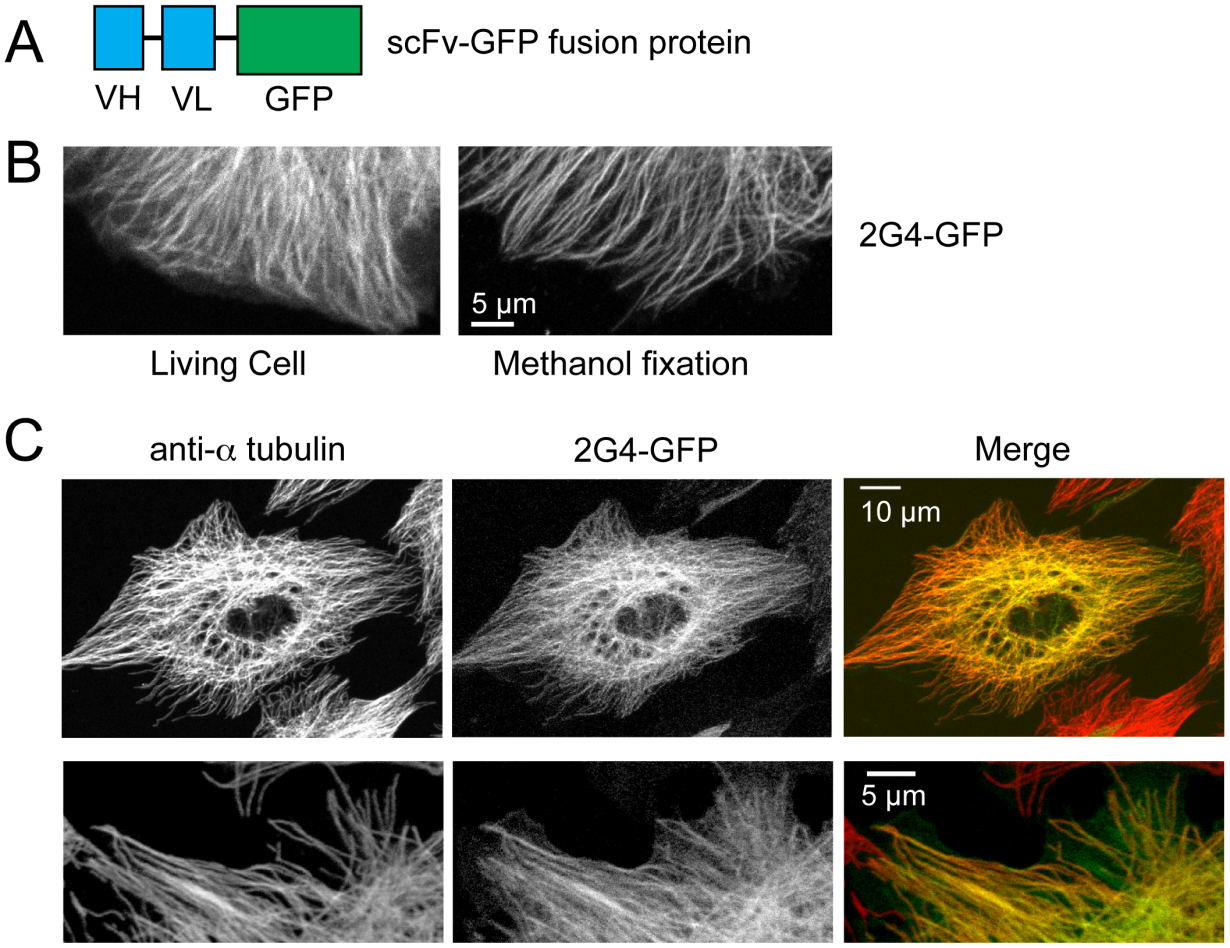
The scFv 2G4-GFP colocalizes with the majority of microtubules. (A) Diagram outlining the recombinant antibody. The combined mw of the V_H_ and V_L_ regions approximately equals that of EGFP. (B) 2G4-GFP localizes to linear filaments in either living cells or in cells fixed in −20°C methanol. Images shown are from the edges of two different LLCPK cells (scale bar = 5 µm). (C) 2G4-GFP localizes to the majority of microtubules. LLCPKs were transfected with plasmid encoding 2G4-GFP and fixed 24 h later. Microtubules were stained with an antibody to a-tubulin. Images in the top row show a maximum intensity projection from a Z series (scale bar = 10 µm). The bottom row shows single optical section from the edge of a second LLCPK cell (scale bar = 5 µm).

Previously we reported that an anti-tubulin scFv, 2G4, can be expressed as a GFP fusion protein in human cell lines [7]. We confirmed those observations and found that 2G4-GFP was expressed as a soluble protein in LLCPK cells (Figure 1 A,B), Hela (not shown) and mouse embryo fibroblast cells (not shown). In each of these cell lines 2G4-GFP localized in a radial pattern that extended to the cell periphery (Figure 1B,C). After methanol fixation and staining with an anti- α-tubulin antibody (antibody B512 targeting α-tubulin’s C terminus (see Methods)), we found that 2G4-GFP co-localized with nearly all microtubules, seen most clearly at the cell periphery, and localized uniformly along the lengths of microtubules (Figure 1C). For some cells, 2G4-GFP was also present in the nucleus, but this must represent free 2G4-GFP since tubulin was not detected here by conventional antibody localizations.

**Figure 2:**
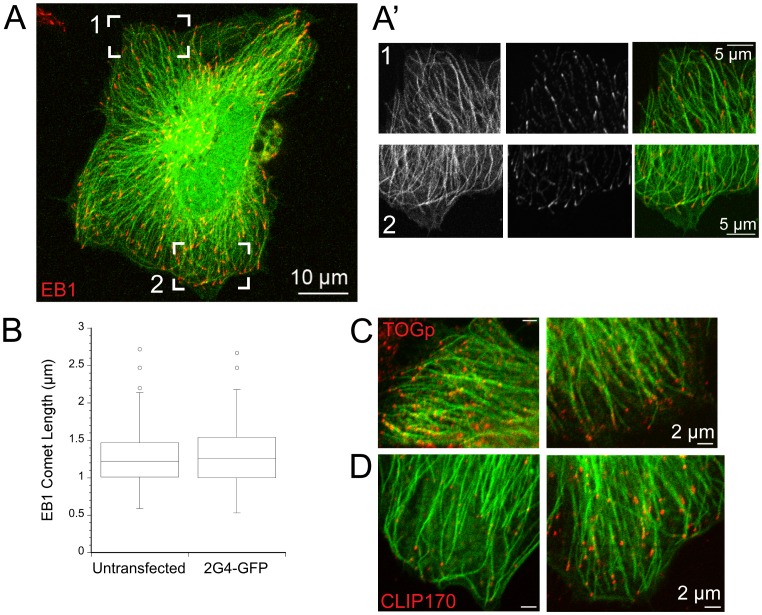
Expression of scFv 2G4-GFP does not disrupt protein binding to microtubule plus ends. (A) 2G4-GFP binding extends to the distal ends of microtubules, as marked by EB1. A maximum intensity projection from optical sections through a Hela cell is shown (scale bar = 10 µm). (A′) Single optical sections from bracketed regions of the cell shown in (A) (scale bar = 5 µm). 2G4-GFP binds uniformly along microtubules and extends to the ends of these microtubules. (B) Box plot of EB1 comet lengths at microtubule plus ends. Expression of 2G4-GFP did not change the length of EB1 comets. (C) TOGp, another microtubule plus end binding protein, is localized to microtubules labeled by 2G4-GFP. (D) CLIP-170 was also localized to microtubule plus ends in cells expressing 2G4-GFP. Note that CLIP-170 was localized to only a subset of microtubule ends. We observed a similar pattern in Hela cells expressing GFP- a-tubulin (data not shown). Scale bars in C, D = 2 µm.

We confirmed that 2G4-GFP bound to the distal ends of microtubules by co-labeling 2G4-GFP expressing cells with an antibody to end binding protein 1 (EB1), a protein that localizes in a comet-like shape to the distal ∼1 µm of growing microtubules [29]. We elected to examine EB1 over other microtubule plus end binding proteins because EB1 binds directly to microtubule plus ends, while several other plus end binding proteins are at least partially dependent on EB1 for their localization to microtubules [29], [30]. Hela cells were fixed approximately 24 h after transfection and stained with an antibody recognizing EB1. In cells expressing 2G4-GFP, EB1 was bound to the ends of many microtubules, consistent with binding to the population of growing microtubule ends (Figure 2A). The 2G4-GFP signal extended to the distal end of EB1 labeled comet, at least to within the ∼200 nm resolution of conventional fluorescence microscopy. The length of individual EB1 comets on microtubule ends was also not affected by 2G4-GFP expression (Figure 2B). Observations in living LLCPK cells co-expressing 2G4-GFP and mCherry-EB3 also showed that 2G4-GFP binding extended to the distal tip of the microtubule (not shown). These data demonstrate that 2G4-GFP binds along microtubules, extending at least to within 200 nm of the microtubule end and that 2G4-GFP does not interfere with EB1 binding to microtubule ends.

Recent data has shown that TOGp binds distal to EB1 at microtubule plus ends [31]. Therefore, we also co-labeled 2G4-GFP expressing Hela cells with an antibody to TOGp. Consistent with the results from EB1 labeling, TOGp was found at the distal ends of most 2G4-GFP labeled microtubules (Figure 2C). A third plus end binding protein, CLIP-170 was also localized to a subset of microtubule plus ends in Hela cells (Figure 2D). This localization pattern was not the result of 2G4-GFP expression since we observed a similar localization pattern in cells expressing GFP- α-tubulin (data not shown). Taken together, these data indicate that 2G4-GFP binds to microtubules and extends to their distal ends without disrupting the binding of several plus end binding proteins.

A second anti-tubulin scFv, 2F12-GFP, was present as aggregates when expressed in Hela or LLCPK cells, consistent with our previous results for expression of this scFv in mammalian cells [7]. Here we found that expression of 2F12-GFP in Hela cells did not disrupt the microtubule cytoskeleton and that tubulin did not colocalize with the 2F12-GFP clusters (Figure S1). These data indicate that 2F12-GFP does not bind and aggregate tubulin dimers when expressed in human cell lines, presumably because the aggregated scFv is inactive. Given these results, we did not explore possible use of this scFv as either a microtubule marker, or as a tool to disrupt microtubule functions in living cells.

### 2G4-GFP is a Reliable Biosensor for Microtubule Dynamic Instability in Living Cells

**Figure 3:**
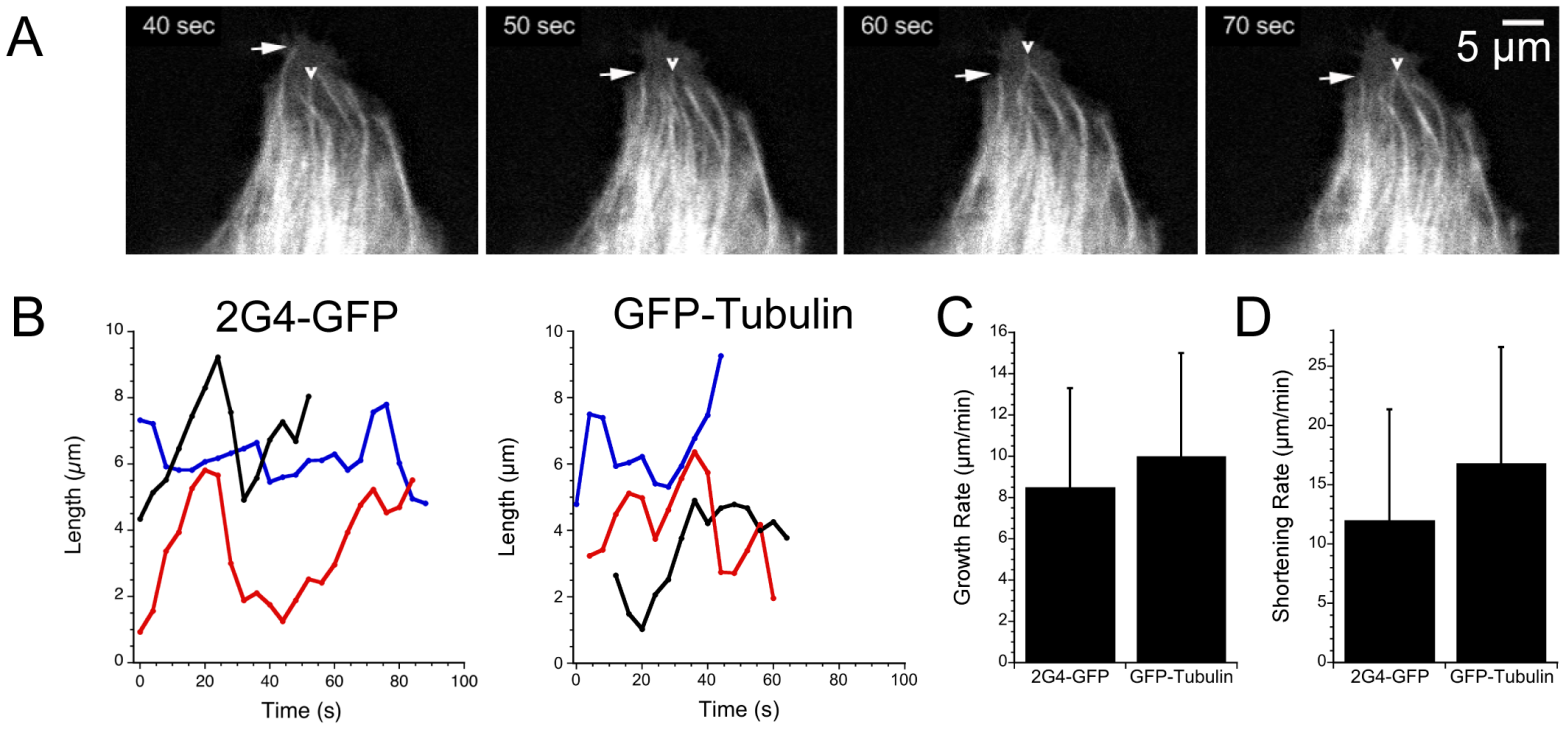
Microtubule length changes detected by 2G4-GFP or GFP- α-tubulin. (A) Sequential images from the periphery of an LLCPK cell expressing 2G4-GFP. Arrows note length changes of several microtubules. Scale bar = 5 µm. The video sequence is presented in Movie S1. (B) Plots of microtubule length changes over time for three microtubules labeled by 2G4-GFP or by GFP-α-tubulin. Length changes over time were determined from image series as described in Methods (C,D). Microtubule growth (C) and shortening velocities (D) measured by 2G4-GFP or GFP-α-tubulin. Data shown are means ± sd. Additional parameters of dynamic instability are summarized in Table 1.

Microtubule dynamic instability has been well characterized in the LLCPK cell line [20], therefore we expressed 2G4-GFP in these cells to determine whether expression of this scFv could serve as an exogenous tag for tubulin without disrupting microtubule assembly dynamics. Living cells were imaged over time (2 - 5 s intervals for 1 - 3 m total time per cell). As shown in Figure 3A,B, the plus ends of the microtubules, as marked by 2G4-GFP, showed the growth and shortening states characteristic of dynamic instability (See Movie S1). We also followed microtubule assembly in an LLCPK cell line stably expressing GFP-α-tubulin [20]. The positions of microtubule ends were tracked and plots of microtubule lengths over time are shown in Figure 3B for 2G4-GFP and GFP-α-tubulin labeled microtubules. The parameters of dynamic instability measured using 2G4-GFP labeling were strikingly similar to those measured in cells expressing GFP-α-tubulin (Figure 3C,D) and to previously published values for this cell line (Table 1). These comparisons indicate that the binding of 2G4-GFP to microtubules did not disrupt microtubule dynamics.

**Table 1:** Comparison of microtubule dynamic instability measured by EGFP-tubulin or 2G4-GFP expression in LLCPK cells.

Dynamic Instability Parameters	2G4-GFP	EGFP-Tub	EGFP-Tub [20]	EGFP-Tub [18]
Growth Rate (µm/min)	8.5±4.8	10.0±5.0	11.5±7.4	8.5±5.8
Shortening Rate (µm/min)	12.0±9.4	16.9±9.9	13.1±8.4	11.3±7.9
Catastrophe Frequency (s^−1^)	.027±.004	.05±.01	.026±.024	.053±.003
Rescue Frequency (s^−1^)	.086±0.13	.106±.024	.175±.104	.086±.005
Percent Time in growth, shorteningand pause	63.7, 19.3, 27	54.2, 26.6, 19.2	15, 11.5, 73.5	40.4, 26, 36.5
Dynamicity (µm/min)	8.2±5.6	9.34±5.5	4±3.5	5.3±2.7

Parameters of dynamic instability measured using EGFP-α-tubulin (EGFP-Tub) as a tracer, expressed at about 10% of total a-tubulin levels, compared to that measured using 2G4-GFP bound to the external surface of microtubules. Published data sets derived from LLCPK cells expressing EGFP-α-tubulin are also included for comparison. Cells measured in reference [18] also expressed CFP.

### 2G4-GFP Binds with Relatively Weak Affinity to Microtubules

The localization of 2G4-GFP to microtubules, without a measurable impact on microtubule dynamics, stimulated us to ask whether 2G4-GFP bound tightly to microtubules, as one might expect of an antibody, or with a relatively weak affinity, similar to MAPs. As a first estimate of 2G4-GFP binding to microtubules, we measured fluorescence intensity along single microtubules at the distal edges of LLCPK cells and compared these values to the intensity of adjacent microtubule-free cytosol. The intensity of 2G4-GFP bound to microtubules was 1.7 times (±0.4, 29 microtubules in 10 cells) brighter than the adjacent cytoplasm. Given that microtubules take up a small percentage of cell volume, these measurements provide a qualitative indication that most of the 2G4-GFP expressed in a cell is not bound to microtubules, and therefore this scFV may bind only weakly to microtubules.

**Figure 4:**
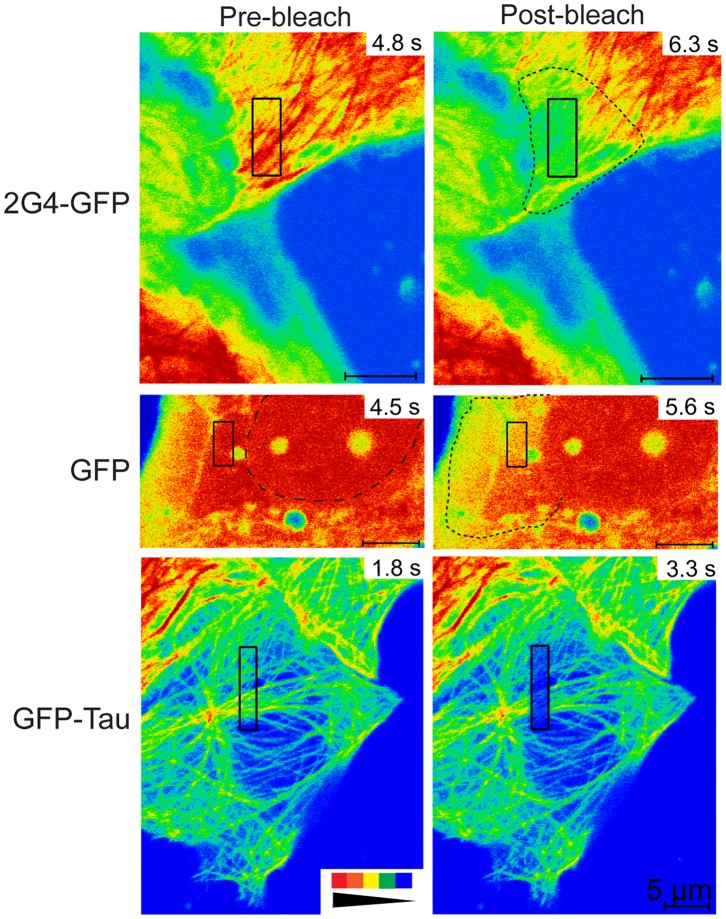
Photobleached 2G4-GFP spreads beyond the region targeted by the bleaching laser. Pre-bleach and post-bleach images are shown for LLCPK cells expressing 2G4-GFP, GFP or GFP-tau as noted. Pre-bleach images were collected immediately prior to photobleaching an area marked by the black rectangular boxes. Post-bleach images were collected immediately after photobleaching. Time in each frame is given in seconds from the start of an imaging experiment. Fluorescence intensity is represented by a rainbow palette from red to blue (see inset at bottom left). For both 2G4-GFP and GFP, the region of dimmed fluorescence has spread beyond that targeted by the bleaching laser. This spread is highlighted by dotted lines in the post-bleach images. The spread of photobleached proteins is likely due to rapid diffusion in and out of the photobleached area. For the GFP pre-bleach image, the nucleus is outlined by a dashed line. Note that fluorescence within the nucleus does not appear to exchange significantly with that in the cytoplasm over the ∼2 s interval between images. In contrast to 2G4-GFP and GFP, GFP-tau photobleaching yields an area of dimmed fluorescence that closely matches the region targeted by the laser. Scale bars for all images are 5 µm.

To estimate 2G4-GFP binding affinity for microtubules within living cells, we used fluorescence recovery after photobleaching (FRAP) to measure 2G4-GFP turnover. We first measured fluorescence recovery within photobleached rectangular shapes positioned near the periphery of cells or in regions closer to the nucleus. Images of cells collected immediately after photobleaching of 2G4-GFP did not yield a distinct photobleached region corresponding to the rectangular area targeted by the laser. Instead, the bleached molecules were distributed over a greater area centered on the rectangular target (Figure 4). One mechanism that could spread the bleached molecules outside the original target area would be diffusion of 2G4-GFP during the brief time interval required for photobleaching and capture of the first image immediately after the bleaching scans. To determine whether a soluble protein would show a spread of photobleaching molecules during the time scale of bleaching and image collection, we photobleached GFP expressed in LLCPK cells. As shown in Figure 4, photobleached GFP was detected well beyond the region targeted by the photobleaching laser. In contrast to GFP or 2G4-GFP, photobleached GFP-tau was detected primarily in the region targeted by the laser (Figure 4).

**Figure 5:**
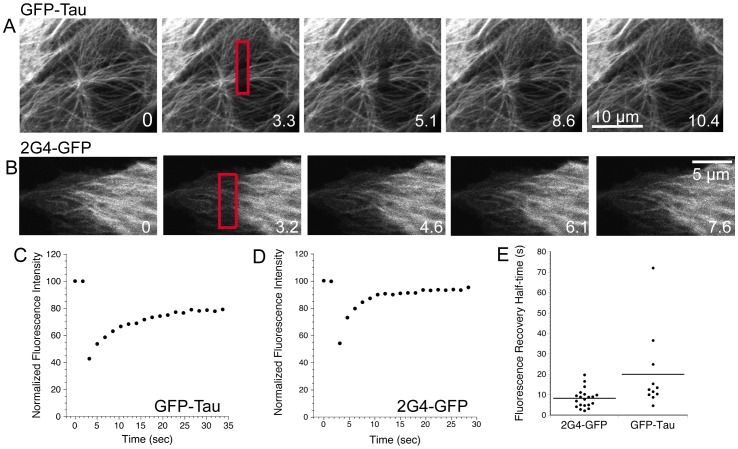
Rapid recovery of GFP-tau or 2G4-GFP after fluorescence photobleaching. (A,B) Images of GFP-tau or 2G4-GFP expressing LLCKP cells before (time 0) and after photobleaching rectangular boxes (bleached regions indicated in red) are shown. Time is given in s. (C,D) Typical fluorescence recovery curves for photobleached rectangles in cells expressing GFP-tau or 2G4-GFP. Fluorescence was normalized as described in Methods. (E) Dot plot showing the half times of fluorescence recovery within boxed regions for 2G4-GFP or GFP-tau. Video sequences are available as Movie S2, S3, S4.

Based on the first images recorded immediately after photobleaching, it appeared that a large fraction of 2G4-GFP was free to move rapidly within the cytoplasm. To extend this observation, we measured fluorescence recovery within the photobleached rectangular areas. Fluorescence recovery over time reflects both diffusion of soluble 2G4-GFP and 2G4-GFP turnover on microtubules. 2G4-GFP fluorescence quickly recovered after photobleaching (Figure 5; see also Movie S2, S3). The average half time of recovery for 2G4-GFP was 8.3±4.6 s (90.9±7%), indicating rapid exchange of 2G4-GFP into and out of the photobleached area. This rapid recovery rate was independent of the position of the bleached area within the cell and was much faster than the published rate of fluorescent tubulin recovery after photobleaching of interphase microtubules (average 200 s; [32]). These data indicate that 2G4-GFP is turning over on the microtubule lattice (see below) and is not simply entering the bleached area on polymerizing microtubules.

For comparison, we expressed and photobleached GFP-tau (isoform with three microtubule-binding repeats). Photobleaching of GFP-tau in rectangular patterns of approximately the same size as those used to photobleach 2G4-GFP resulted in more clearly defined photobleached regions visible in images immediately after photobleaching (Figure 4, 5; see also Movie S4). GFP-tau fluorescence recovered with a half time of 20±19 s (86.5±11% recovery) (see Figure 5 B–E). This recovery half time is similar to measurements made in fibroblasts [33], including recovery on single microtubules and microtubule bundles (see Table 2). Comparison of recovery half times for photobleached GFP-tau and 2G4-GFP indicated that turnover of 2G4-GFP was faster than that observed for GFP-tau (p = 0.014). In each case, recovery of fluorescence within the boxed area likely includes both diffusion of soluble protein and turnover of microtubule-bound proteins.

**Table 2:** 2G4-GFP turns over on microtubules at a rate similar to MAPs.

Protein	Half time of fluorescence recovery on microtubules	Reference
2G4-GFP	4.5 ± 4.3 s	this study
GFP-tau	6.8 ± 4 s	this study
GFP-tau	2.3 ± 0.4 s	[33]
Ensconsin	4.3 s	[37]
EB1-GFP	3.6 - 12 s	[38]
CLIP-170	< 1 s	[39]

Fibroblast data is shown for GFP-tau [33]. Ensconsin turnover was measured by expression of its microtubule-binding domain fused to 5 GFPs [37]. EB1-GFP turnover on microtubule ends was measured in both mitotic and interphase Xenopus egg extracts [38]. Half times of recovery are given as mean ± SD, or as described in the publications cited.

**Figure 6:**
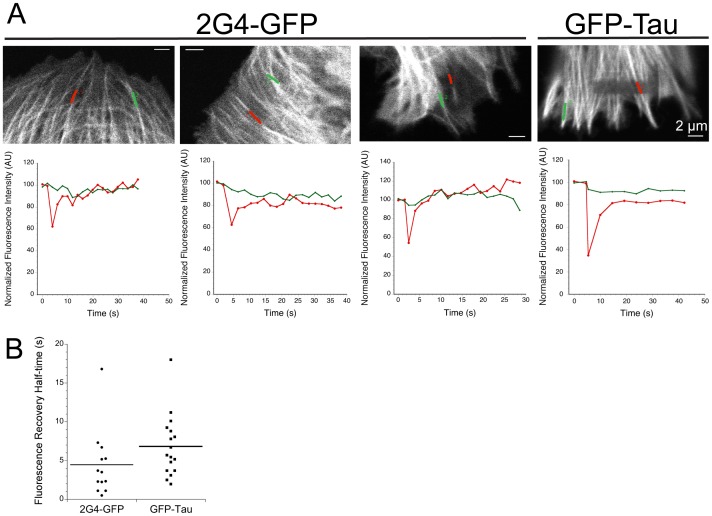
2G4-GFP turns over rapidly on microtubules. (A) Images shown were recorded immediately after photobleaching. Line scans along individual photobleached microtubules within LLCPK cells expressing either 2G4-GFP or GFP-tau are positioned as indicated. Line scan 1 (red) follows a microtubule region within the photobleached area and line scan 2 (green) follows a microtubule outside the photobleached rectangle. Normalized fluorescence recovery, integrated over each line, is shown below each example. (B) Dot plot showing fluorescence recovery half times.

To confirm that the rapid turnover estimated within the photobleached areas included rapid turnover of the fraction of GFP-tagged proteins bound to microtubules, we also measured fluorescence intensity along individual microtubules (Figure 6A) during the recovery phase. Three examples are shown for 2G4-GFP fluorescence bound to microtubules within (red line) and outside the bleached zone (green line) (Figure 6A). In each case, fluorescence recovers within seconds along the surface of the microtubule (Figure 6A). Quantitation of recovery rates yielded a half time of 4.5±4.3 s for 2G4-GFP on microtubules (89.7±11.8% recovery, 13 microtubules) (see Figure 6B). The recovery rate measured for microtubule-bound GFP-tau was similar to that measured for 2G4-GFP (half-time 6.8±4 s; 95.8±6.5% recovery; 15 microtubules; Figure 6) and the rates of fluorescence recovery on microtubules for GFP-tau and 2G4-GFP were not significantly different (p = 0.13). The high percentages of recovery for GFP-tau and 2G4-GFP on microtubules indicates that nearly all fluorescent proteins were able to exchange with their respective soluble pools. Taken together, the FRAP analysis demonstrates that 2G4-GFP binds to microtubules with an affinity similar to GFP-tau.

**Figure 7:**
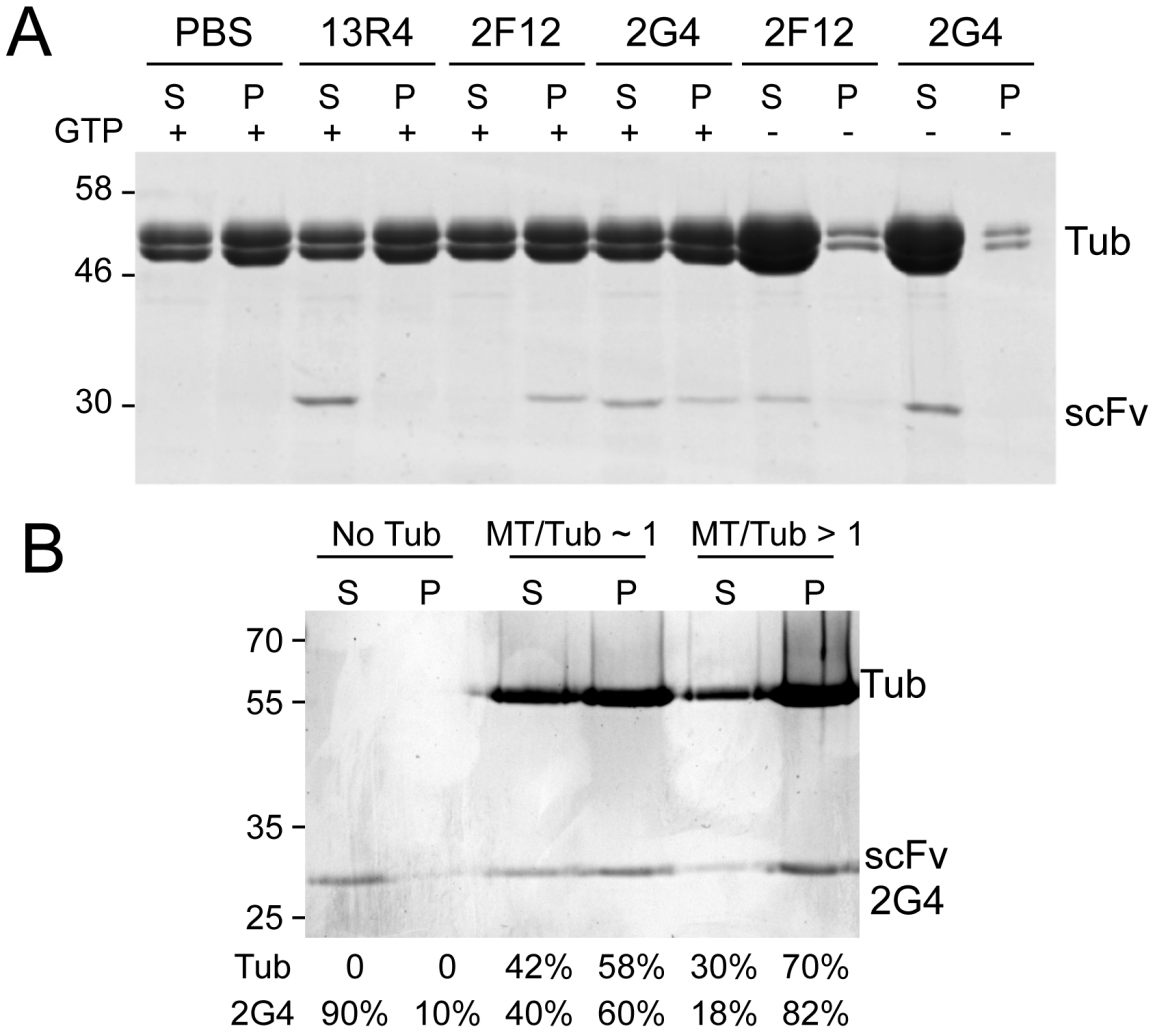
Purified 2G4 co-pellets with microtubules. Purified porcine brain tubulin was polymerized by addition of GTP and warming to 37°C. After incubation with purified scFv’s, the microtubule fraction was isolated by pelleting through a 40% sucrose cushion and the supernatants and pellets resolved on SDS-PAGE gels. The two anti-tubulin scFv’s, 2F12 and 2G4, co-pelleted with microtubules, but a greater fraction of 2F12 was pelleted compared to 2G4, consistent with comparatively weaker binding of 2G4 to microtubules. 13R4, an anti-ß-galactosidase scFv, did not co-pellet with microtubules, indicating that scFv are not trapped in the microtubule pellet. To confirm that co-pelleting represents binding to microtubules, GTP was omitted from the assembly mixture to significantly reduced tubulin polymerization. Under these conditions 2F12 and 2G4 are found in the supernatant fraction. B) To confirm that the fraction of scFv 2G4 present in the supernatant was active, we stabilized polymerized microtubules with Taxol in order to obtain a higher ratio of microtubules to soluble tubulin. The same experiment described in (A) was repeated in the absence (labeled “No Tub”), and in the presence of about equal concentrations of microtubules and tubulin (labeled “MT/Tub∼1”) and most tubulin polymerized into microtubules (labeled “MT/Tub>1”). The bands were quantified and the percentage of soluble tubulin, microtubules, and soluble and pelleted scFv are indicated below the lanes. Because of saturation of the Coomassie signal, tubulin quantitations are approximate and the percentage of tubulin in the pellet fraction is underestimated in the last lane.

The cell-based measurements reported above indicated that 2G4-GFP binds microtubules with an affinity similar to MAPs. Co-pelleting of purified 2G4 with in vitro assembled porcine brain microtubules supported the idea that 2G4 binds with relatively weak affinity for microtubules (Figure 7A). Three scFv’s were tested for their ability to co-pellet with microtubules: an anti-ß-galactosidase scFv 13R4, and two anti-tubulin scFv, 2F12 and 2G4. 13R4 remained in the soluble fraction and did not co-pellet with microtubules. In contrast, both 2F12 and 2G4 co-pelleted with microtubules. This co-pelleting represents binding to microtubules since neither scFv was found in the pellet fraction when GTP was omitted from the assembly mixture, which significantly reduced tubulin polymerization. Under standard microtubule assembly conditions, 2F12 co-pelleted with microtubules with very little 2F12 remaining in the supernatant fraction. In contrast, 2G4 was distributed in both supernatant and pellet fractions (Figure 7A). To exclude the possibility that a fraction of 2G4 is inactive and therefore unable to co-pellet with microtubules, Taxol (a microtubule stabilizing drug) was added to the reaction mixture, which increased the amount of tubulin in the pellet fraction. As shown in Figure 7B, inclusion of Taxol increased the amount of tubulin in the pellet fraction and more than 80% of the purified 2G4 scFv was able to bind microtubules. These data demonstrate that purified 2G4 is active, but binds microtubules with a relatively weaker affinity compared to the scFv, 2F12.

### 2G4-GFP Recognizes Tyrosinated α-tubulin

**Figure 8:**
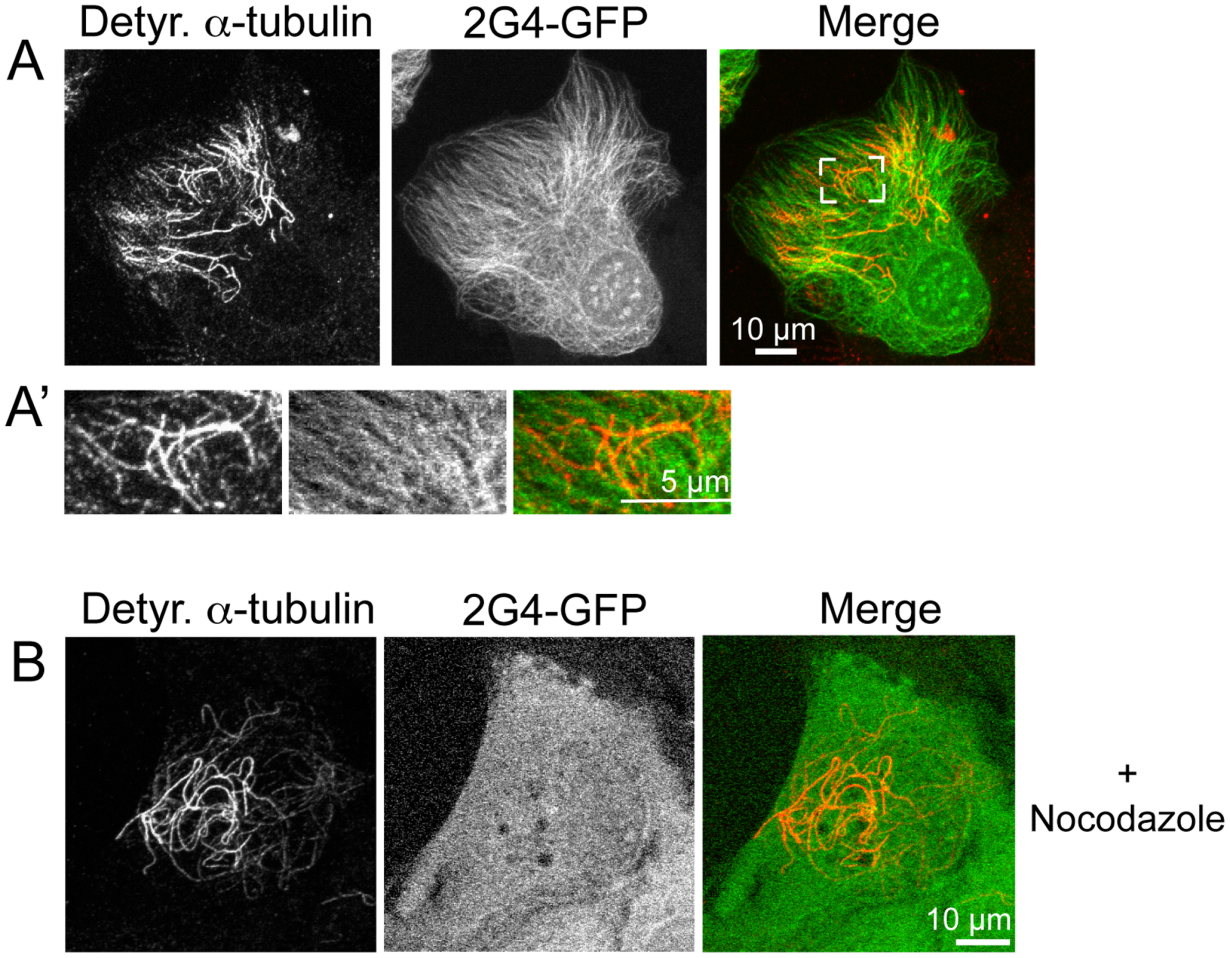
Recombinant scFv 2G4-GFP does not co-localize with de-tyrosinated α-tubulin. (A) LLCPK cells were fixed 24 h after transfection and stained with antibodies specific for de-tyrosinated α-tubulin. The area bracketed in white is enlarged in (A′). 2G4-GFP does not show detectable binding to microtubules recognized by an antibody specific for detyrosinated α-tubulin. (B) LLCPK cells expressing 2G4-GFP were incubated in 33 µM nocodazole for 15 m prior to fixation and localization of detyrosinated α-tubulin. Depolymerization of the majority of microtubules shifted 2G4-GFP to a soluble protein present uniformly throughout the cell and it did not colocalize with microtubules composed of detyrosinated α-tubulin. Scale bars = 10 µm (whole cell images) and 5 µm (enlarged region). Images shown are maximum intensity projections from optical sections.

Non-dynamic microtubules often comprise only a small subset of the total microtubule population within rapidly dividing cells grown in culture [8], [13]. Therefore, we also asked whether 2G4-GFP co-localized with either of two common post-translational modifications to a-tubulin: detyrosination or acetylation. Note that detyrosination and acetylation can mark separate subpopulations of microtubules in the same cell [14]. As shown in Figure 8A and A′, 2G4-GFP and anti-detyrosinated a-tubulin recognized non-overlapping subsets of microtubules in fixed LLCPK cells. These observations were repeated in a second cell line (MEFs; not shown). To confirm that 2G4-GFP did not bind to microtubules composed of detyrosinated tubulins, LLCPK cells were incubated in 33 µM nocodazole for 15 min prior to fixation. Under these conditions the dynamic microtubules depolymerize, but the more stable microtubules remain. As shown in Figure 8B, 2G4-GFP was present uniformly throughout the cell and did not colocalize with the detyrosinated microtubules.

Microtubules marked by tubulin acetylation were also examined. Acetylated microtubules were typically more abundant than detyrosinated microtubules in LLCPK cells and the 2G4-GFP signal showed overlap with a subset of the microtubules recognized by anti-acetylated α-tubulin in fixed LLCPK cells (Figure S2).

**Figure 9:**
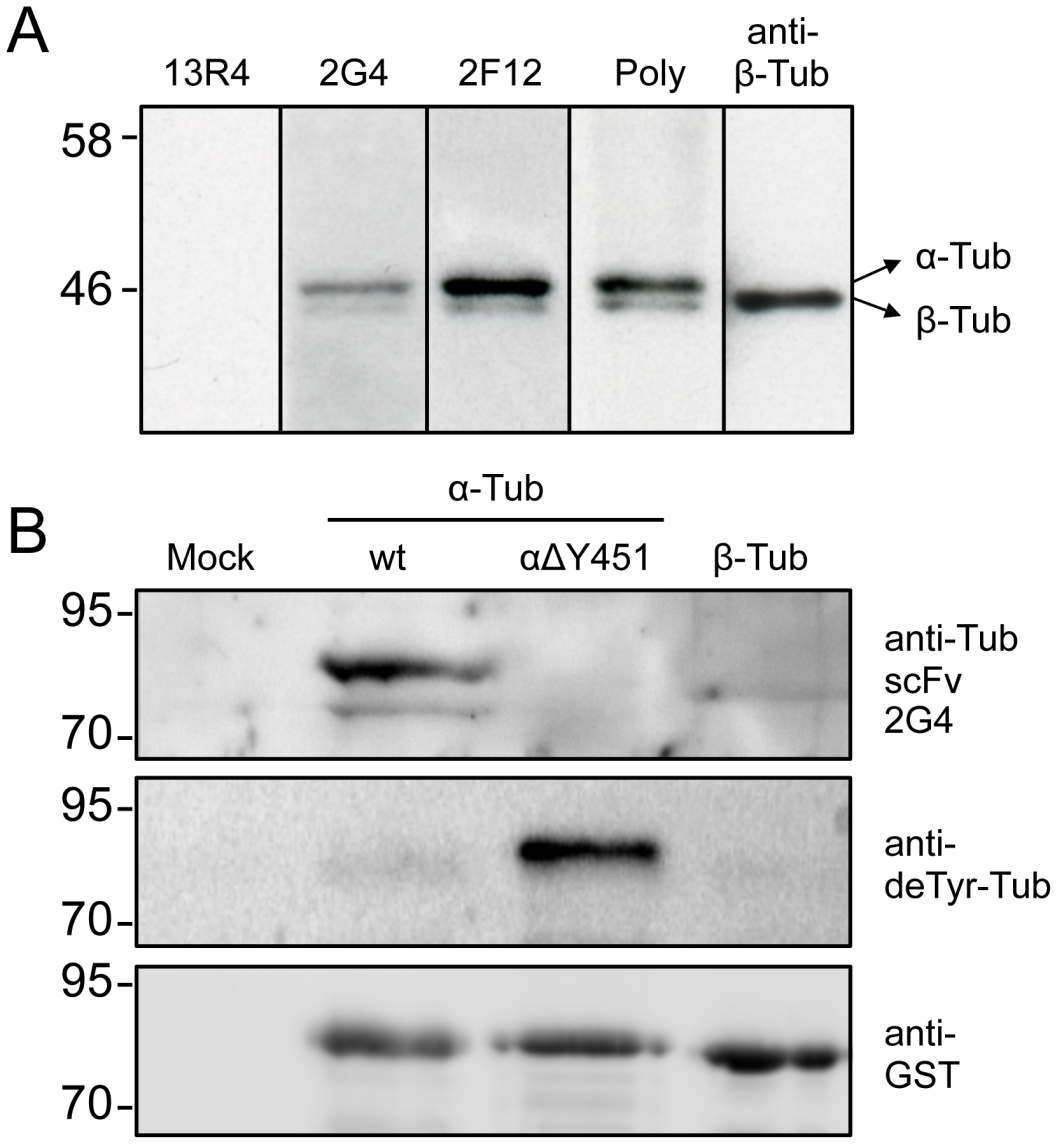
Recombinant antibody 2G4 recognizes tyrosinated, but not detyrosinated α-tubulin. (A) Immunoblot of pig brain extracts probed with scFv’s or anti-tubulin antibodies. Results are shown for the purified recombinant antibodies 13R4 (irrelevant anti-ß-galactosidase scFv), 2G4 or 2F12 as described in Methods. Signals from commercial anti-tubulin polyclonal and monoclonal anti-ß-tubulin antibodies are shown for comparison. (B) Total E. coli extracts from cells expressing GST-tubulin fusion proteins were probed with the scFv 2G4 (10 µg/ml), anti-detyrosinated tubulin, or with an anti-GST antibody (loading control). The terminal tyrosine of α-tubulin is deleted from the delta Y451 fusion protein. See methods section for sequence accession numbers.

Based on the lack of 2G4-GFP binding to detyrosinated-a-tubulins in LLCPK cells, we asked whether 2G4 recognizes α- or ß-tubulin isolated from pig brain and whether a-tubulin’s C terminal tyrosine was necessary for scFv binding. By immunoblot, purified His-tagged 2G4 recognized porcine brain α-tubulin, with only minimal recognition of ß-tubulin (Figure 9A). To address whether binding requires α-tubulin’s terminal tyrosine, GST-tagged α-tubulin, including or missing the C-terminal tyrosine residue were expressed in E. coli. By immunoblotting, purified 2G4 recognized GST- α-tubulin and this binding was abolished by deletion of α-tubulin’s C-terminal tyrosine residue (Figure 9B). Thus, 2G4-GFP recognizes tyrosinated, but not de-tyrosinated, α-tubulin. In addition, 2G4 was specific to α-tubulin and only a slight band was visible in the ß-tubulin-GST extract (Figure 9B). This band may reflect either a weak cross-reactivity to ß-tubulin, as observed in blots of brain extracts (Figure 9A), or weak non-specific binding to an E. coli protein. Given that the GST antibody does not recognize two bands (Figure 9B), it is unlikely that the lower α-tubulin represents a proteolytic fragment.

## Discussion

Here we described an scFv, 2G4, tagged with GFP, that is soluble when expressed in mammalian cells, colocalizes with microtubules and binds microtubules with an affinity similar to that measured for several microtubule-associated proteins. We posit that the weak binding affinity is sufficient to allow localization, without disrupting microtubule function, as measured here by microtubule assembly dynamics and the binding of EB1, TOGp and CLIP-170 to microtubule ends. For study of the microtubule cytoskeleton, expression of 2G4-GFP provides an alternative to expression of GFP-tagged α-tubulin. Although all evidence to date indicates that the GFP tag does not compromise α-tubulin function, there may be situations where an alternative tag that is not assembled as part of the microtubule could be advantageous. For example, the GFP at the N-terminus of α-tubulin should be positioned within the lumen of the microtubule where it could possibly interfere with studies of tubulin acetylation, a modification localized to the inside surface of the hollow microtubule [34]. More generally, we posit that scFV that bind relatively weakly to their antigen could prove useful as intracellular biosensors by labeling, but not disrupting, a dynamic structure. Another example of a weak-binding exogenous tag is Lifeact, a 17 amino acid polypeptide that binds F-actin. Expression of Lifeact-GFP marks actin filaments in living cells without compromising actin functions [35]. For more stable structures, higher affinity scFv’s may also provide useful markers of protein localization in live cells.

Typical screens have focused on identifying high affinity antibodies or scFv (e.g. [36]), but the results presented here demonstrate that relatively weak binding of an scFv can be advantageous for development of intracellular biosensors. 2G4-GFP bound to microtubules with an affinity comparable to several MAPs [33], [37]–[39], as measured by the large cytoplasmic pool of 2G4-GFP, and the rapid recovery of fluorescence after photobleaching. For the MAP ensconsin, the K_d, apparent_ for microtubules was estimated to be ∼11 µM [37], and we assume that 2G4-GFP binds microtubules with an affinity in this range, given their similar recovery rates (the recovery rate constant is equal to the dissociation constant, k_off_, as described in [37]). We did not attempt to use additional fluorescence measurements to estimate the K_d, apparent_ of 2G4-GFP binding to microtubules because it is likely that 2G4-GFP binds to both free tubulin dimers and to microtubule polymers. Evidence for binding soluble tubulin dimers is based on the ability of 2G4 to recognize tubulin by immunoblot (Figure 9). Therefore, 2G4-GFP is not in a simple equilibrium between microtubule-bound and unbound states. Instead, 2G4-GFP should be in a complex with either soluble tubulin dimers or tubulins assembled into microtubules. Despite binding both soluble tubulin and microtubules, 2G4-GFP did not disrupt microtubule dynamic instability, and it is therefore likely that 2G4-GFP also binds weakly to tubulin dimers.

The other interesting feature of the 2G4 scFv is that it is able to differentiate between α-tubulins differing only in the presence or absence of the C-terminal tyrosine residue. 2G4 colocalizes with, and binds to, tyrosinated α-tubulin, but not detyrosinated α-tubulin (Figure 1, 8, 9). The C-terminus of tubulin is located on the exterior surface of the microtubule [40], where it is accessible to 2G4-GFP. Some, but not all, microtubules containing acetylated α-tubulin were recognized by 2G4-GFP. Acetylation occurs on α-tubulin’s lysine-40 and this amino acid is located on the inner lumen of the microtubule [9] where it is unlikely that an scFv would have access. Acetylation within the microtubule lumen is thought to alter the conformation of the microtubule on the outer surface [34], [41] and this altered conformation could reduce 2G4 binding to the microtubule’s outer surface. Overall, the ability of 2G4 to discriminate between tyrosinated and detyrosinated a-tubulins demonstrates the feasibility of isolating intracellular soluble scFv specific for particular post-translational modifications, which could allow live cell imaging of these specific protein modifications.

In summary, here we describe use of a recombinant antibody as an intracellular tracer, specific for one form of a protein and able to differentiate between unmodified and post-translational protein modifications. Intracellular expression of fluorescently tagged scFv, combined with treatments to enrich for a post-translational modification, should allow isolation of biosensors able to detect specific protein post-translational modifications in living cells.

## Supporting Information

Figure S1 **The scFv 2F12 is insoluble in mammalian cells.** A second anti-tubulin scFv, 2F12, was not soluble when expressed in LLCPK cells. This scFv forms aggregates that are independent of tubulin. The microtubule cytoskeleton is not disrupted in cells expressing 2F12. Scale bar = 10 µm.
(TIF)

Figure S2 **2G4-GFP recognizes acetylated microtubules.** LLCPK cells were fixed 24 h after transfection and stained with antibodies specific for acetylated a-tubulin. 2G4-GFP labeled microtubules show some co-localization with those recognized by an anti-acetylated α-tubulin antibody. (B). Arrows in the enlarged region show microtubules co-labeled by 2G4-GFP and anti-acetylated α-tubulin. Scale bars = 10 µm (whole cell images) and 5 µm (enlarged regions).
(TIF)

Movie S1 **Microtubule length changes marked by binding of 2G4-GFP.** Individual frames from this video are shown in Figure 3A. Time is given in minutes:seconds. Scale bar = 5 µm.
(M4V)

Movie S2 **2G4-GFP recovers rapidly after photobleaching.** The region marked by the red box was photobleached at 3.2 seconds. Time is given in seconds. Scale bar = 2 µm. Frames from this video sequence are shown in Figure 5B.
(M4V)

Movie S3 **Second example of 2G4-GFP recovery after photobleaching.** The region marked by the red box was photobleached at 0.105 minutes. Time is given in minutes, scale bar = 2 µm.
(M4V)

Movie S4 **Tau-GFP recovery after photobleaching.** The region marked by the red box was photobleached at 3.3 seconds. Time is given in seconds, scale bar = 2 µm. Frames from this video sequence are shown in Figure 5A.
(M4V)
