# Supplementary figures and images for: Specific In Vivo Labeling of Tyrosinated α-Tubulin and Measurement of Microtubule Dynamics Using a GFP Tagged, Cytoplasmically Expressed Recombinant Antibody
Source: PLoS One. 2013 Mar 28;8(3):e59812. doi: 10.1371/journal.pone.0059812 (PMC3610906; doi:10.1371/journal.pone.0059812)

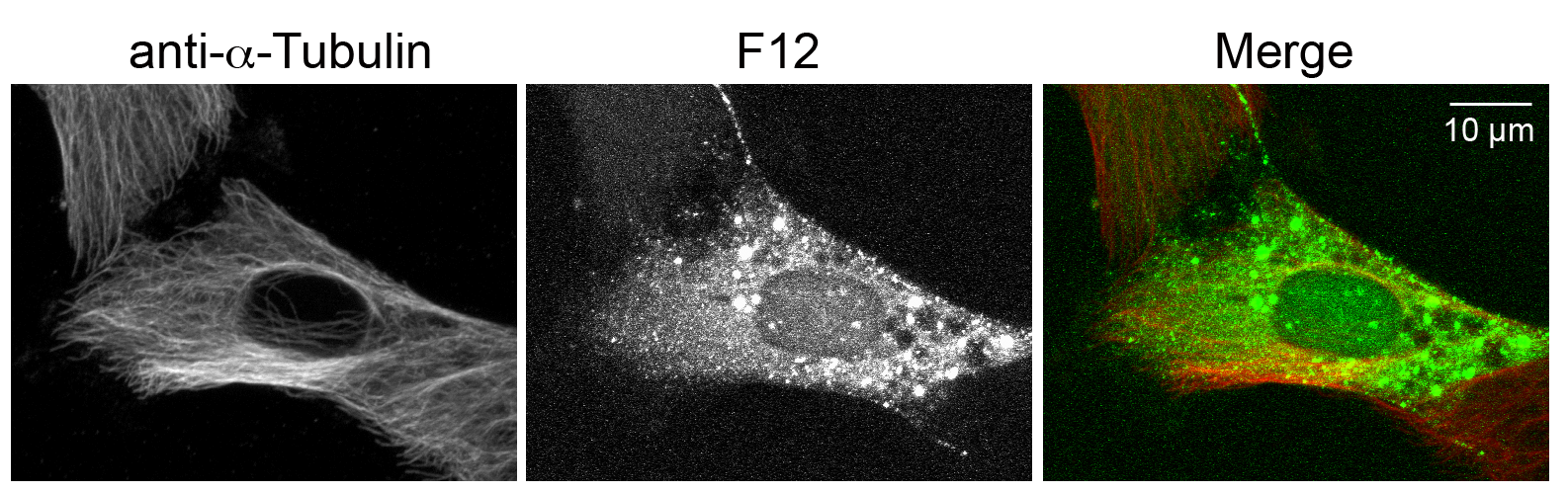

Supplement: Figure S1 — The scFv 2F12 is insoluble in mammalian cells. A second anti-tubulin scFv, 2F12, was not soluble when expressed in LLCPK cells. This scFv forms aggregates that are independent of tubulin. The microtubule cytoskeleton is not disrupted in cells expressing 2F12. Scale bar = 10 µm. (TIF) [file pone.0059812.s001.tif]

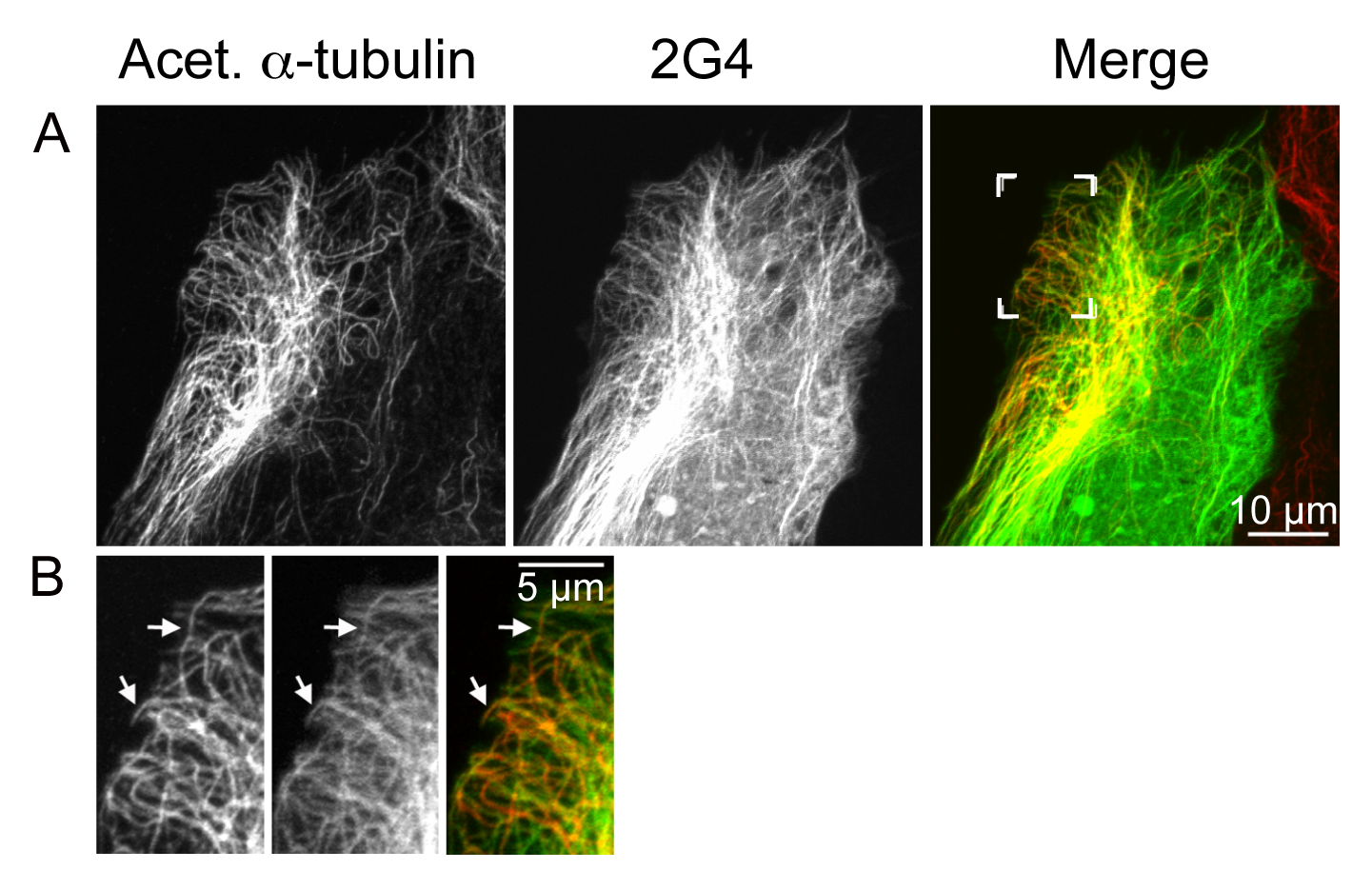

Supplement: Figure S2 — 2G4-GFP recognizes acetylated microtubules. LLCPK cells were fixed 24 h after transfection and stained with antibodies specific for acetylated a-tubulin. 2G4-GFP labeled microtubules show some co-localization with those recognized by an anti-acetylated α-tubulin antibody. (B). Arrows in the enlarged region show microtubules co-labeled by 2G4-GFP and anti-acetylated α-tubulin. Scale bars = 10 µm (whole cell images) and 5 µm (enlarged regions). (TIF) [file pone.0059812.s002.tif]
